# Application of a *Burkholderia cepacia *lipase-immobilized silica monolith to batch and continuous biodiesel production with a stoichiometric mixture of methanol and crude Jatropha oil

**DOI:** 10.1186/1754-6834-4-42

**Published:** 2011-10-21

**Authors:** Koei Kawakami, Yasuhiro Oda, Ryo Takahashi

**Affiliations:** 1 Department of Chemical Engineering, Faculty of Engineering, Graduate School, Kyushu University, 744 Moto-oka, Nishi-ku, Fukuoka 819-0395, Japan

## Abstract

**Background:**

The enzymatic production of biodiesel through alcoholysis of triglycerides has become more attractive because it shows potential in overcoming the drawbacks of chemical processes. In this study, we investigate the production of biodiesel from crude, non-edible Jatropha oil and methanol to characterize *Burkholderia cepacia *lipase immobilized in an *n*-butyl-substituted hydrophobic silica monolith. We also evaluate the performance of a lipase-immobilized silica monolith bioreactor in the continuous production of biodiesel.

**Results:**

The Jatropha oil used contained 18% free fatty acids, which is problematic in a base-catalyzed process. In the lipase-catalyzed reaction, the presence of free fatty acids made the reaction mixture homogeneous and allowed bioconversion to proceed to 90% biodiesel yield after a 12 hour reaction time. The optimal molar ratio of methanol to oil was 3.3 to 3.5 parts methanol to one part oil, with water content of 0.6% (w/w). Further experiments revealed that *B. cepacia *lipase immobilized in hydrophobic silicates was sufficiently tolerant to methanol, and glycerol adsorbed on the support disturbed the reaction to some extent in the present reaction system. The continuous production of biodiesel was performed at steady state using a lipase-immobilized silica monolith bioreactor loaded with 1.67 g of lipase. The yield of 95% was reached at a flow rate of 0.6 mL/h, although the performance of the continuous bioreactor was somewhat below that predicted from the batch reactor. The bioreactor was operated successfully for almost 50 days with 80% retention of the initial yield.

**Conclusions:**

The presence of free fatty acids originally contained in Jatropha oil improved the reaction efficiency of the biodiesel production. A combination of *B. cepacia *lipase and its immobilization support, *n*-butyl-substituted silica monolith, was effective in the production of biodiesel. This procedure is easily applicable to the design of a continuous flow-through bioreactor system.

## Background

Production of biodiesel (fatty acid alkyl esters) through transesterification of virgin plant oils, as well as low-quality waste oils with short-chain alcohols, has received considerable attention during the past decade for producing a biodegradable and nonpolluting fuel. The conventional method for industrial biodiesel production is a chemical process using acid or base catalysts [1-3]. The chemical process offers a high yield of biodiesel in a short reaction time but has drawbacks, such as the need to remove the catalyst and by-products, and high energy consumption [1-5]. As an alternative, an enzymatic process using lipase as a biocatalyst may overcome these problems because lipases can catalyze a variety of transesterification and esterification reactions relatively efficiently under mild conditions and in non-aqueous environments [1-8]. Thus considerable research has been directed at achieving high yields of biodiesel in short reaction times, for various feedstocks ranging from virgin vegetable oils to low-quality acid oils with high free fatty acid content. Among non-edible oils, *Jatropha curcas*, which is toxic owing to the presence of carcinogenic phorbol esters, has great potential for biodiesel production [9-13].

Immobilization of lipase is an essential technology in enabling us to perform continuous production of biodiesel using packed-bed bioreactors [14-21]. The immobilized *Candida antarctica *B lipase commercialized as 'Novozyme 435' has been investigated widely and is reported to exhibit the best performance [22-24]. However, although methanol is the most commonly used acyl acceptor, the enzyme is deactivated in the presence of small droplets of insoluble methanol [15,22,25]. Several strategies have been attempted to overcome this adverse effect, including stepwise addition of methanol to the reaction mixture [15,22,26], use of alcohols with longer alkyl chains than methanol [24,26-30], use of methyl or ethyl acetate [23,31-33] as acyl acceptors, and the introduction of organic solvents, such as *ter*-butanol, that are not accessible to the lipase [18,26,34-38]. Other researchers have shown that lipases from *Pseudomonas *species are the most promising for biodiesel production [9,27-29,39-45]. Immobilization support materials include particles of diatomaceous earth [9,40,42], polypropylene [40,42,44,45], mesoporous silica [45], kaolinite [27], and silica-polyvinyl alcohol composite [43,46].

It is now well known that lipases are highly activated and stabilized, owing to conformational change leading to the open-lid structure, when encapsulated in alkyl-substituted silicates by the sol-gel method [47,48], and the resultant preparations are used effectively for many organic syntheses [49-51]. Several researchers have applied sol-gel immobilized lipases to the production of biodiesel. Hsu *et al*. [16,28,30] prepared immobilized *Burkholderia cepacia *(formerly *Pseudomonas cepacia*) lipase in a phyllosilicate sol-gel matrix and used it for the transesterification of tallow and grease. Repeated production of ethyl esters was also performed in a recirculating packed-column bioreactor loaded with particles of immobilized lipase [16]. Noureddini *et al*. [41] immobilized *B. cepacia *lipase within a hydrophobic *iso*-butyl-substituted sol-gel support and succeeded in producing methyl and ethyl esters yield of 67% and 65%, respectively, from soybean oil in 1 hour. Lipase from the same origin was also immobilized in a methyl-substituted silica aerogel and applied in the transesterification of sunflower seed oil with methyl acetate [32].

Recently, we demonstrated that a macroporous, non-shrinkable silica monolith could be formed easily from a mixture of methyltrimethoxysilane (MTMS) and tetramethoxysilane (TMOS), and that an enzyme-immobilized silica monolith was applicable as a flow-through microbioreactor for organic syntheses [52]. We also developed a highly efficient bioreactor loaded with a lipase-immobilized silica monolith by adopting a two-step sol-gel method, that is, by preparing an MTMS-based silica monolith coated with butyl-substituted silicates that entrapped lipase [53]. We applied this type of bioreactor to the continuous production of fatty acid methyl esters through methanolysis of rapeseed oil in *n*-hexane by *Rhizopus oryzae *lipase [54]. The use of such an enzyme-immobilized silica monolith bioreactor is expected to be useful for the biodiesel production, because it offers several benefits, including very low backpressure, high contacting efficiency and mechanical durability, as compared with conventional packed-bed bioreactors [52-54]. In the present study, we selected *B. cepacia *lipase as the most promising enzyme, and investigated the production of biodiesel through solvent-free methanolysis of Jatropha oil in batch and continuous bioreactors loaded with lipase-immobilized silica monoliths.

## Results and Discussion

### Batchwise production of biodiesel using a lipase-immobilized crushed silica monolith

#### **Characterization of methanolysis of crude Jatropha oil by lipase-immobilized silicates**

The crude Jatropha oil used was received as a kind gift from the Hak plantation, Banteay Meanchey, Cambodia. The fatty acid composition of this oil is given in Table 1. The composition of the four acids, palmitic, stearic, oleic and linoleic, was in the range of previously reported values [12,13]. However, the oil originally contained 18% free fatty acids, possibly owing to inappropriate handling and storage. This caused a miscible solution at a stoichiometric 3:1 molar mixture of methanol and Jatropha oil. The rapeseed oil used was fatty acid-free and did not form a miscible solution with methanol.

**Table 1:** Fatty acid composition of the Jatropha oil

Fatty acid	Structure	% (w/w)
Palmitic	16:0	14.6
Palmitoleic	16:1	0.7
Stearic	18:0	6.9
Oleic	18:1	46.2
Linoleic	18:2	30.8
Linolenic	18:3	0.2
Arachidic	20:0	0.3
Non-identified		0.3

Free fatty acids		18.3
Monoglyceride		2.1
Diglyceride		12.1
Triglyceride		65.8

**Figure 1:**
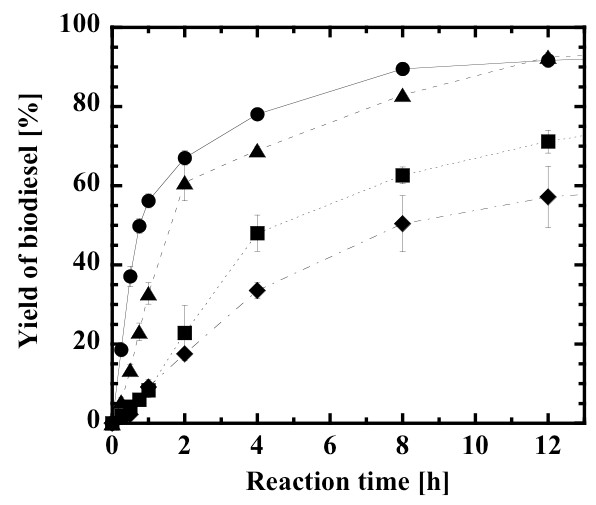
**Comparison of the production rate of biodiesel from different oil feedstocks**. Jatropha oil and methanol (closed circles), rapeseed oil and methanol (closed squares), rapeseed oil, 18% oleic acid and methanol (closed triangles), and Jatropha oil and methanol with non-immobilized lipase (closed diamonds). Methanol:oil molar ratio, 3:1; water content, 0.6% (w/w).

In this study, the molar ratio of methanol to oil was determined based on the molar amount of triglyceride that was supposed to be originally present in the feedstock. Therefore the 3:1 molar mixture meant an equimolar mixture of methanol and total fatty acids contained in the feedstock. Figure 1 compares the rate of biodiesel production from different oil feedstocks. The methanol to oil molar ratio of 3:1 was selected because the production rate was highest at this molar ratio in the early stage of the conversion. The rate of methanolysis of rapeseed oil was low, probably owing to the insoluble nature of methanol in the rapeseed oil, causing direct contact between methanol droplets and lipase molecules and thereby deactivating the lipase. The methanolysis rate of crude Jatropha oil containing 18% free fatty acid was the highest among the systems investigated, and the biodiesel yield approached nearly 90% after 12 hours. In addition, the immobilized lipase gave a six-fold higher initial conversion rate as compared with its non-immobilized counterpart, being consistent with previous study [47-51,53,54]. The methanolysis of rapeseed oil containing 18% oleic acid was attempted to examine the effect of homogeneity of the reaction mixture. Interestingly, the reaction rate increased and exhibited similar kinetic behavior to the methanolysis of crude Jatropha oil. An additional reason for the increase in production rate may be that the rate of methyl-esterification of fatty acids is generally faster than that of the methanolysis of triglycerides [55,56]. Jachmanian *et al*. [20] have also reported that the addition of alkyl esters appears to be a useful tool to ensure homogeneous conditions in both substrate and product mixtures. In any case, this trend illustrates an advantage of the lipase method in the production of biodiesel, because in the alkaline method, incorporation of the free fatty acid leading to soap formation should be avoided.

#### Effect of water content on biodiesel yield

**Figure 2:**
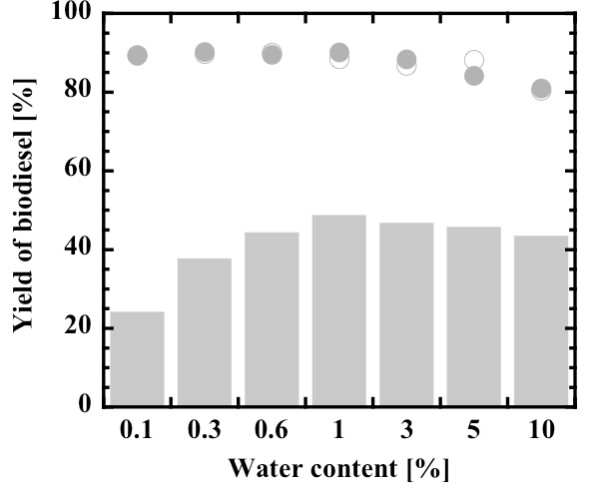
**Effect of water content on biodiesel yield**. After 0.5 hours (closed gray bars), after 12 hours (open circles) and after 24 hours (closed gray circles). Methanol:oil molar ratio, 3:1.

Water content is an important parameter in bioconversions in non-aqueous media. A small amount of water is required to maintain conformational flexibility of the lipase molecules, while excess water causes ester hydrolysis [7,8,27,29,41,44]. Figure 2 shows the effect of water content, ranging from 0.1% to 10% (w/w) on the basis of total mass of reaction mixture, on the biodiesel yield in the methanolysis of Jatropha oil. The biodiesel yield at 0.5 hours increased with an increase in water content up to 1% and decreased slightly thereafter. The final yields of biodiesel at 12 hours and 24 hours were influenced less by an increase in water content up to 1%, but decreased gradually with further increase in water content. This suggests that the biodiesel production proceeded at a water content up to 1% without being subjected to hydrolysis reaction. The water content of 1% (w/w) is equivalent to 0.8% reaction volume. Shah and Gupta [9] reported that 0.7% (v/v) was optimum in the ethanolysis of Jatropha oil by immobilized *B. cepacia *lipase. In this study, a water content of 0.6% was applied in subsequent experiments, because water contents of more than 1% (w/w) became insoluble in the initial reaction mixture.

#### Effect of molar ratio of methanol to oil on biodiesel yield

**Figure 3:**
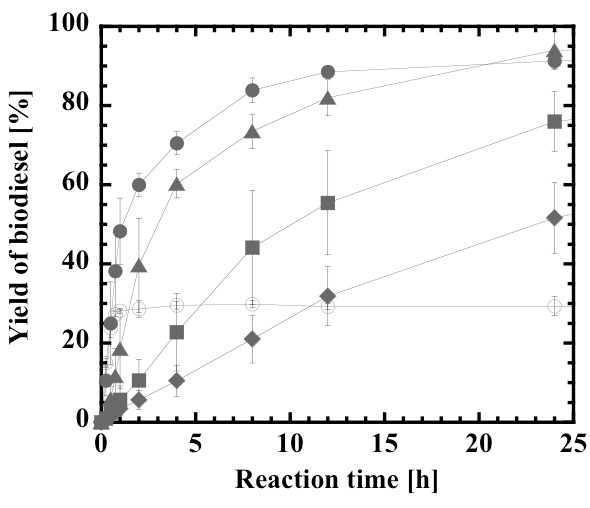
**Effect of methanol:oil molar ratio on the production rate of biodiesel**. 1:1 (open circles), 3:1 (closed circles), 4:1 (closed triangles), 5:1 (closed squares), and 6:1 (closed diamonds). Water content, 0.6% (w/w).

Although the stoichiometric molar ratio of the reaction is 3:1, all the lipases are more or less subjected to inhibition and inactivation by alcohols, especially methanol. The optimal molar ratio is therefore dependent on the lipase type. Figure 3 shows the rate of biodiesel production at the different molar ratios of methanol to oil ranging from 1:1 to 6:1. The biodiesel production rate was highest at the stoichiometric molar ratio of 3:1 and decreased sharply with a further increase in molar ratio up to 6:1. A detailed investigation revealed that the optimal molar ratio was 3.3 to 3.6:1, as described in later section.

A three-step separate addition of methanol was attempted at 0 hours, 0.5 hours and 1 hour, to investigate the extent of lipase inactivation by methanol. No significant difference was observed from the one-step whole addition at the start of reaction (data not shown). This agrees with the result reported by Shah and Gupta [9] that *B. cepacia *lipase has much greater resistance to methanol as compared with other lipases.

#### Effect of glycerol on the production of biodiesel

**Figure 4:**
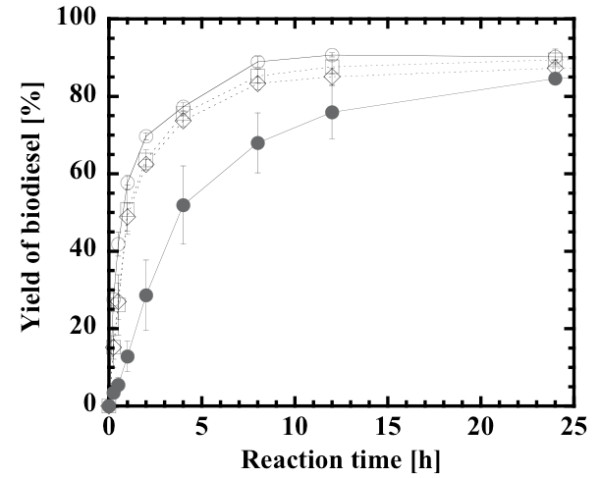
**Effect of washing of used immobilized lipase during its repeated use**. First reaction (open circles), second reaction after washing with acetone (open squares), second reaction after washing with *ter*-butanol (open diamonds), second reaction without washing (closed circles). Methanol:oil molar ratio, 3:1; water content, 0.6% (w/w).

Glycerol, a by-product of alcoholysis, is physically adsorbed on the surface of immobilized lipase, retards the mass transfer of oil to lipase active sites and accordingly lowers the rate of biodiesel production. In the present system involving the stoichiometric mixture of methanol and oil, 10% (w/w) glycerol is formed on the basis of the total mass of reaction mixture, when the reaction is completed. The effect of the external addition of glycerol up to 10% (w/w) was investigated. As a result, the initial production rate of biodiesel was decreased by approximately 30% with increasing glycerol concentration up to 10% (w/w), as compared with no addition of glycerol (data not shown). *ter*-Butanol can solubilize glycerol, and is often used as a solvent in lipase-catalyzed biodiesel production [18,26,34-38]. The immobilized lipase used for the 24 hour reaction was washed with *ter*-butanol or acetone and reused in the second cycle of the same reaction. Figure 4 compares the rate of biodiesel production by the fresh catalyst in the first cycle and non-washed and washed catalysts in the second cycle. Although the initial activity of the non-washed catalyst in the second cycle decreased by 20% compared with that of the fresh catalyst, that of the washed catalyst was restored by 60% compared with that of the fresh catalyst. It was, however, apparent that the biodiesel yields after a 24 hour reaction time converged within the range of 85% to 90%.

#### Effect of addition of silica gel on the biodiesel yield

**Figure 5:**
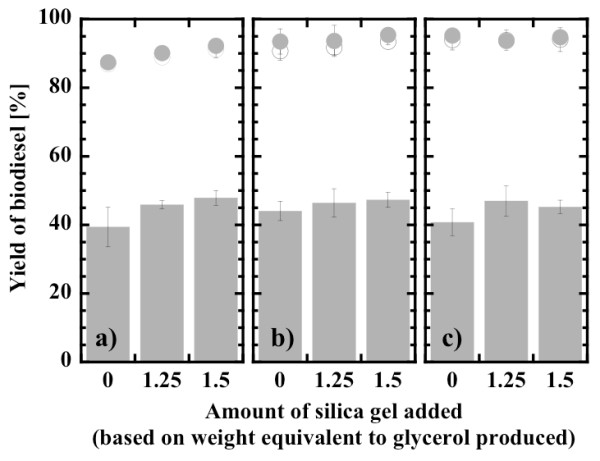
**Effect of the addition of silica gel on biodiesel yield**. After 0.5 hours (closed gray bars), after 12 hours (open circles) and after 24 hours (closed gray circles). Methanol:oil molar ratio, (a) 3:1; (b) 3.3:1; and (c) 3.5:1. Water content, 0.6% (w/w).

A maximum yield of 90% was attained after 12 hours at the stoichiometric molar ratio of 3:1. Stevenson *et al*. [57] reported that the final yield of biodiesel could be further increased by adding silica gel, which may adsorb the glycerol more strongly than the immobilized lipase particles. It has also been reported that the silica gel adsorbs the methanol and its addition is effective for designing a prolonged release system for methanol [58]. The effect of silica gel addition on biodiesel yield was thus investigated for the molar ratio of methanol to oil. The effect of the latter was examined again within the narrow range of molar ratios between 3:1 and 3.5:1. The amount of silica gel added was determined to be 1.25 and 1.5 weight equivalents compared with the amount of glycerol produced when the 3:1 stoichiometric mixture was completely reacted. As shown in Figure 5, the results were rather complex. The biodiesel yields after 0.5 hours tended to be slightly increased with an increase in silica gel at the three molar ratio levels, although they differed in extent. No significant effect of molar ratio was observed on the biodiesel yields after 0.5 hours. These results probably mean that part of the glycerol produced was favorably removed from the surface of the immobilized lipase, but methanol inhibition was of minor influence. The final biodiesel yields after 12 hours and 24 hours were also slightly increased by the addition of silica gel at molar ratios of 3:1 and 3.3:1, whereas they were unchanged at a molar ratio of 3.5:1 giving the highest yield of approximately 95%. It is also noteworthy that the final biodiesel yields were increased up to 95% at the maximum with an increase in molar ratio of 3:1 to 3.5:1. A possible explanation for the latter may be that a small loss of methanol by vaporization and/or strong distribution into a separated glycerol phase was compensated for by the slight increase in the initial amount of methanol.

### Application of a lipase-immobilized silica monolith bioreactor to the continuous production of biodiesel

**Figure 6:**
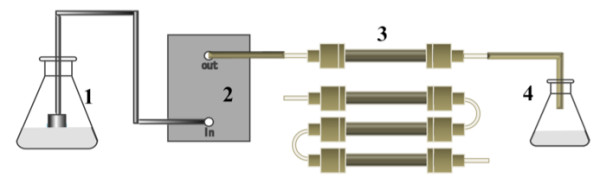
**Schematic representation of a flow-through silica monolith bioreactor system**. (1) Reservoir for substrate solution, (2) semi-micro HPLC pump, (3) lipase-immobilized silica monolith bioreactor (10 mm × 10 cm or 30 cm), and (4) receiver for product solution.

**Figure 7:**
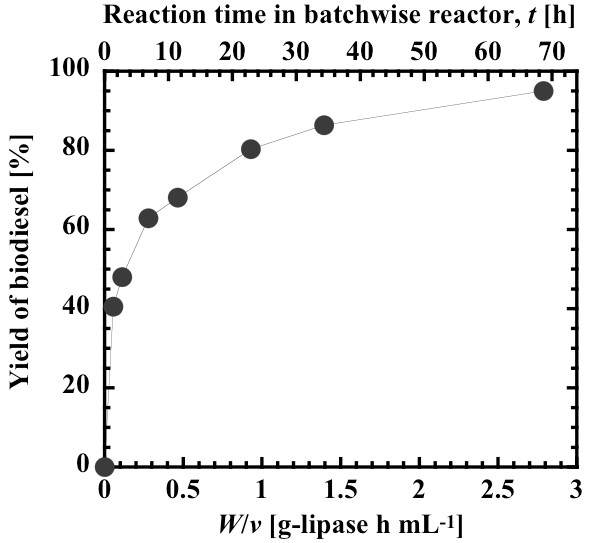
**Steady state yields of biodiesel in the lipase-immobilized silica monolith bioreactor**. The yields are shown as a function of *W*/*v *(mass of lipase divided by flow rate of substrate solution). The substrate solution, composed of methanol and oil at 3:1 molar ratio and 0.6% (w/w) water, was fed continuously at constant flow rates between 0.6 mL/h and 30 mL/h to the bioreactor (10 mm × 30 cm) loaded with 4.92 g silica monolith containing 1.67 g lipase.

Silica monolith columns are characterized by mechanical durability of the support and low backpressure of the substrate solution flowing through a macroporous structured support, as compared with conventional packed beds. A lipase-immobilized silica monolith bioreactor was applied to the continuous production of biodiesel from crude Jatropha oil. Figure 6 shows a schematic representation of the bioreactor system used for flow-through reaction experiments. The stock solution comprised a 3:1 stoichiometric mixture of methanol and oil and 0.6% (w/w) water. The molar composition of 3:1 was selected for comparison with the majority of the experimental data collected using a non-steady state batchwise reactor. The three lipase-immobilized silica monolith columns (10 mm inside diameter, 10 cm length) were connected in series and the substrate solution was allowed to pass through the resultant bioreactor at a volumetric flow rate of 0.6 mL/h to 30 mL/h. Figure 7 shows the relation between the biodiesel yield and time variable, *W*/*v *(mass of lipase present in the bioreactor divided by the volumetric flow rate of substrate solution) under steady state operation. If the substrate solution flows through the bioreactor in ideal plug flow, the time variable is equivalent to *mt*/*V *(product of the liquid volume based-mass concentration of lipase and reaction time) in a batch slurry reactor [21,53,54]. The reaction time, *t*, calculated from the equation:

t=WV∕mv

is given on the upper horizontal axis of Figure 7. The continuous monolith bioreactor exhibited lowered biodiesel yields, as compared with the batchwise slurry reactor (Figures 1, 3 and 5). In previous studies on lipase-catalyzed transesterifications in organic solvents, the performance of the continuous silica monolith microbioreactor was superior to that of the batchwise slurry reactor in terms of conversions at the same values of the time variables, *W*/*v *and *mt*/*V *[53,54]. The decrease in yield in the present biodiesel production system may be a result of the high viscosity of the substrate solution that prevents a uniform forced flow through interparticle spaces within the monolith. Another reason may be steady state adsorption of glycerol on the surface, of which amount may be larger than that in a non-steady state batch reaction. However, the biodiesel yield at steady state under the conditions of lowest volumetric flow rate (0.6 mL/h, *W*/*v *= 2.79 g-lipase h/mL) reached 95%.

**Figure 8:**
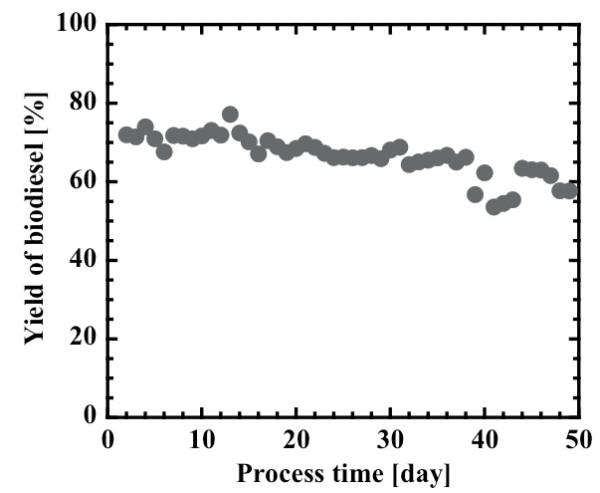
**Operational stability of the lipase-immobilized silica monolith bioreactor in the continuous production of biodiesel**. The substrate solution, composed of methanol and oil at 3:1 molar ratio and 0.6% (w/w) water, was fed continuously at a constant flow rate of 0.06 mL/h to the bioreactor (10 mm × 10 cm) loaded with 1.87 g silica monolith containing 0.63 g lipase.

As shown in Figure 8, the operational stability of the lipase-immobilized silica monolith bioreactor was tested by a continuous flow of the substrate solution for a longer period of time at a fixed *W*/*v *value of 1.05 g-lipase h/mL. Although the biodiesel yield gradually decreased from 73% to 58% after 49 days of operation, 80% of the initial yield was retained even after continuous operation for almost 50 days. Washing of the monolith was attempted by replacing the substrate solution with *ter*-butanol during the operation. However, the steady state biodiesel yield after washing was not returned to the initial yield, giving the same yield as that right before washing. It is therefore probable that a gradual decrease in yield resulted from slow inactivation of the lipase by methanol.

## Conclusions

An *n*-butyl-substituted silica monolith that immobilized *B. cepacia *lipase was applied successfully to the production of biodiesel through the methanolysis of crude Jatropha oil containing 18% free fatty acid. A biodiesel yield of 90% was reached in a 12 hour batch reaction using a stoichiometric mixture of methanol and oil (3:1), with a water content of 0.6% (w/w) and at a temperature of 40°C. The continuous production of biodiesel was also achieved using a lipase-immobilized silica monolith bioreactor. The steady state yield of 95% was attained at the low flow rate of 0.6 mL/h (2.79 g-lipase h/mL) and 80% of the initial yield was retained even after continuous operation for almost 50 days.

## Methods

### Materials

Lipase PS-SD from *B. cepacia *was purchased from Amano Enzyme Inc. (Nagoya, Japan), and was used without further purification. Jatropha oil used was a kind gift from the Hak plantation, Banteay Meanchey, Cambodia. The fatty acid composition of this oil is shown in Table 1. Rapeseed oil was obtained from Riken Nosan-Kako Co. (Fukuoka, Japan). The composition of fatty acids in this triglyceride was reported by the manufacturer to be 4.2% palmitic acid, 61.6% oleic acid, 19.6% linoleic acid and 10.1% linolenic acid. The methyl esters of the major fatty acids were obtained from Sigma Aldrich Japan Co. (Tokyo, Japan) and used to construct calibration curves via gas chromatography. TMOS, MTMS and other chemicals were of reagent grade and were purchased from Tokyo Chemical Industries Co. (Tokyo, Japan). *n*-butyltrimethoxysilane (BTMS) was kindly supplied by Dow Corning Toray Co. (Ichihara, Japan).

### Preparation of a silica monolith derived from a mixture of MTMS and TMOS

A silica monolith was first prepared using the sol-gel method reported previously [52-54]. At room temperature, a mixture of 3.44 mL MTMS and 890 μL TMOS (molar ratio of MTMS to TMOS, 4:1), 1.05 mL distilled water and 45 μL of 40 mM hydrogen chloride were added consecutively to a test tube. After stirring at room temperature for 10 minutes, the homogeneous sol obtained was cooled to 4°C and 9.85 mL of 100 mM phosphate buffer solution (pH 7.5) was added. The molar ratio of silanes to water was 5:100 in this preparation. Gelation was allowed to proceed for 1 day in a container maintained at 23°C. The resulting hydrogel was lyophilized for 1 day. This produced a non-shrinkable MTMS-based silica monolith in a test tube that was then used as a support for immobilization of lipase.

### Preparation of a lipase-immobilized silica monolith coated with butyl-substituted silicates

A 12 mL sol solution comprising a mixture of BTMS and TMOS at a fixed molar ratio of 4:1 was prepared in the presence of lipase using the procedure described above. Homogeneous sol in an amount of 0.8 mL was mixed with 7.2 mL of 100 mM phosphate buffer solution (pH 7.5), and 4 mL of lipase solution (pH 7.5) was then added. A sol mixture containing 1.2 g lipase was slowly poured into the MTMS-based silica monolith formed in the test tube. The liquid-solid mixture was degassed under reduced pressure for 10 minutes to permeate the sol solution into the interstices of the monolith. Gelation was allowed to proceed for 1 day in a container maintained at 23°C. The lipase-immobilized silica monolith, coated with butyl-substituted silicates, was lyophilized for 1 day, then collected and crushed in a mortar. Typically, particles of 0.74 g crushed silica monolith containing 250 mg lipase were used for batch reaction experiments.

### Preparation of a lipase-immobilized silica monolith bioreactor

A 10-cm-long glass tube with inside diameter of 10 mm was used for making a lipase-immobilized silica monolith bioreactor. The glass tube was placed into a test tube and filled with the sol mixture containing MTMS and TMOS, as described above, resulting in the formation of an MTMS-based silica monolith in both the test tube and in the glass tube. In the second step sol-gel coating, the sol mixture of BTMS and TMOS containing 1.2 g of lipase was poured into the MTMS-based silica monolith formed in the test tube, in which the silica monolith-containing glass tube was still embedded. After a series of treatments as described above, the glass tube was retrieved, and the mass of gel inside and outside the glass tube was measured. The mass of the lipase immobilized within the glass tube was calculated by assuming a uniform distribution throughout the gel. The 10 mm × 10 cm glass tube was used as the lipase-immobilized silica monolith bioreactor. The three units prepared simultaneously in separate test tubes were connected in series, and the resultant 30-cm-long bioreactor was also used for flow-through reaction experiments (Figure 6).

### Batch reaction experiments

The molar ratio of methanol to Jatropha oil is an important variable in the biodiesel production. The Jatropha oil used contained 18% free fatty acids of which composition was the same as that of total fatty acids in oil. In this study, the molar ratio of methanol to oil was determined based on the molar amount of triglyceride that was supposed to be originally present in the feedstock. Therefore the 3:1 molar mixture meant an equimolar mixture of methanol and total fatty acids contained in the feedstock.

A typical reaction mixture was prepared by mixing 5 g Jatropha oil and 0.54 g methanol (molar ratio of methanol to oil, 3:1; and water content, 0.6% (w/w) based on the total mass) in 20 mL screw-capped vials. The mixture was stirred at 1400 min^-1 ^on a shaking incubator maintained at 40°C. The reaction was initiated by the addition of 250 mg lipase powder or 0.74 g particles of crushed silica monolith containing 250 mg crude, immobilized lipase. Major variables investigated were feedstock type, the molar ratio of methanol to oil (1:1 to 6:1) and the water content (0.1% to 10% (w/w)). Reactions were carried out in triplicate. Almost all the data were represented as mean ± standard deviation.

### Flow-through reaction experiments

The glass tubes loaded with lipase-immobilized silica monolith were provided with end fittings, attached to a semi-micro HPLC pump (PU610, GL Sciences Co. (Tokyo, Japan)) and immersed in a constant temperature bath maintained at 40°C. The substrate solution was fed at volumetric flow rates of 0.6 mL/h to 30 mL/h using the HPLC pump. Steady state at each flow rate was confirmed when the exit concentration of the fatty acid methyl esters became independent of the process time.

### Long-term operational stability test

The silica monolith bioreactor loaded with 1.87 g silica monolith, containing 0.63 g of immobilized lipase, was operated continuously for 49 days. A 3:1 mixture of methanol to oil containing 0.6% (w/w) water was fed to the inlet of the bioreactor at a constant volumetric flow rate of 0.06 mL/h.

### Sample analysis

Organic samples were analyzed using a gas chromatograph (GC-14 B, Shimadzu Co. (Kyoto, Japan)) equipped with a low polarity capillary column DB-5 (15 m × 0.25 mm inner diameter and 0.5 μm film thickness: Agilent Technologies (Santa Clara, California, USA)). The column oven temperature was initially held at 150°C for 0.5 minutes, then raised to 300°C at a heating rate of 20°C/min, and finally kept at 300°C for 3 minutes. The carrier gas was nitrogen with a flow rate of 1 mL/min. The injector temperature was maintained at 295°C and detection was conducted with a flame ionization detecotor maintained at 300°C. Concentrations of the four fatty acid methyl esters (methyl palmitate, methyl stearate, methyl oleate and methyl linoleate) were quantified using calibration curves prepared by analyzing standard solutions of mixed methyl esters. The biodiesel yield was determined as a ratio of the total concentration of these four methyl esters to the total concentration of corresponding fatty acids in the initial reaction mixture.

## Competing interests

The authors declare that they have no competing interests.

## Authors' contributions

KK proposed and directed the research. YO and RT conducted the experiments. KK, YO and RT discussed the results. YO compiled an early draft and KK wrote the paper. All authors read and approved the final manuscript.
